# ROS-MUSIC toolchain for spiking neural network simulations in a robotic environment

**DOI:** 10.1186/1471-2202-16-S1-P169

**Published:** 2015-12-18

**Authors:** Philipp Weidel, Renato Duarte, Karolína Korvasová, Jenia Jitsev, Abigail Morrison

**Affiliations:** 1 Institute of Advanced Simulation (IAS-6) & Institute of Neuroscience and Medicine (INM-6), Forschungszentrum Juelich, 52425 Juelich, Germany; 2 Faculty of Psychology, Institute of Cognitive Neuroscience, Ruhr-University Bochum, 44801 Bochum, Germany; 3 Simulation Laboratory Neuroscience - Bernstein Facility for Simulation and Database Technology, Institute for Advanced Simulation, Jülich Aachen Research Alliance, Jülich Research Center, Jülich, Germany

## 

Studying a functional, biologically plausible neural network that performs a particular task is highly relevant for progress in both neuroscience and machine learning. Most tasks used to test the function of a simulated neural network are still very artificial and thus too narrow, providing only little insight into the true value of a particular neural network architecture under study. For example, many models of reinforcement learning in the brain rely on a discrete set of environmental states and actions [1]. In order to move closer towards more realistic models, modeling studies have to be conducted in more realistic environments that provide complex sensory input about the states. A way to achieve this is to provide an interface between a robotic and a neural network simulation, such that a neural network controller gains access to a realistic agent which is acting in a complex environment that can be flexibly designed by the experimentalist.

To create such an interface, we present a toolchain, consisting of already existing and robust tools, which forms the missing link between robotic and neuroscience with the goal of connecting robotic simulators with neural simulators. This toolchain is a generic solution and is able to combine various robotic simulators with various neural simulators by connecting the Robot Operating System (ROS) [2] with the Multi-Simulation Coordinator (MUSIC) [3]. ROS is the most widely used middleware in the robotic community with interfaces for robotic simulators like Gazebo, Morse, Webots, etc, and additionally allows the users to specify their own robot and sensors in great detail with the Unified Robot Description Language (URDF). MUSIC is a communicator between the major, state-of-the-art neural simulators: NEST, Moose and NEURON. By implementing an interface between ROS and MUSIC, our toolchain is combining two powerful middlewares, and is therefore a multi-purpose generic solution.

One main purpose is the translation from continuous sensory data, obtained from the sensors of a virtual robot, to spiking data which is passed to a neural simulator of choice. The translation from continuous data to spiking data is performed using the Neural Engineering Framework (NEF) proposed by Eliasmith & Anderson [4]. By sending motor commands from the neural simulator back to the robotic simulator, the interface is forming a closed loop between the virtual robot and its spiking neural network controller.

To demonstrate the functionality of the toolchain and the interplay between all its different components, we implemented one of the vehicles described by Braitenberg [5] using the robotic simulator Gazebo and the neural simulator NEST.

In future work, we aim to create a testbench, consisting of various environments for reinforcement learning algorithms, to provide a validation tool for the functionality of biological motivated models of learning.
